# Aurisin A Complexed with 2,6-Di-*O*-methyl-β-cyclodextrin Enhances Aqueous Solubility, Thermal Stability, and Antiproliferative Activity against Lung Cancer Cells

**DOI:** 10.3390/ijms23179776

**Published:** 2022-08-29

**Authors:** Thanapon Charoenwongpaiboon, Amy Oo, Sutita Nasoontorn, Thanyada Rungrotmongkol, Somdej Kanokmedhakul, Panupong Mahalapbutr

**Affiliations:** 1 Department of Chemistry, Faculty of Science, Silpakorn University, Nakhon Pathom 73000, Thailand; 2 Center of Excellence in Structural and Computational Biology, Department of Biochemistry, Chulalongkorn University, Bangkok 10330, Thailand; 3 Department of Biochemistry, Center for Translational Medicine, Faculty of Medicine, Khon Kaen University, Khon Kaen 40002, Thailand; 4 Ph.D. Program in Bioinformatics and Computational Biology, Graduate School, Chulalongkorn University, Bangkok 10330, Thailand; 5 Natural Products Research Unit, Department of Chemistry and Center for Innovation in Chemistry, Faculty of Science, Khon Kaen University, Khon Kaen 40002, Thailand

**Keywords:** Aurisin A, beta-cyclodextrins, inclusion complex, lung cancer

## Abstract

Aurisin A (AA), an aristolane dimer sesquiterpene isolated from the luminescent mushroom *Neonothopanus nambi*, exhibits various biological and pharmacological effects. However, its poor solubility limits its use for further medicinal applications. This study aimed to improve the water solubility of AA via complexation with β-cyclodextrin (βCD) and its derivatives (2,6-di-*O*-methyl-βCD (DMβCD) and 2-hydroxypropyl-βCD (HPβCD). A phase solubility analysis demonstrated that the solubility of AA linearly enhanced with increasing concentrations of βCDs (ranked in the order of AA/DMβCD > AA/HPβCD > AA/βCD). Notably, βCDs, especially DMβCD, increased the thermal stability of the inclusion complexes. The thermodynamic study indicated that the complexation between AA and βCD(s) was a spontaneous endothermic reaction, and AA/DMβCD possesses the highest binding strength. The complex formation between AA and DMβCD was confirmed by means of FT-IR, DSC, and SEM. Molecular dynamics simulations revealed that the stability and compactness of the AA/DMβCD complex were higher than those of the DMβCD alone. The encapsulation of AA led to increased intramolecular H-bond formations on the wider rim of DMβCD, enhancing the complex stability. The antiproliferative activity of AA against A549 and H1975 lung cancer cells was significantly improved by complexation with DMβCD. Altogether, the satisfactory water solubility, high thermal stability, and enhanced antitumor potential of the AA/DMβCD inclusion complex would be useful for its application as healthcare products or herbal medicines.

## 1. Introduction

**Figure 1:**
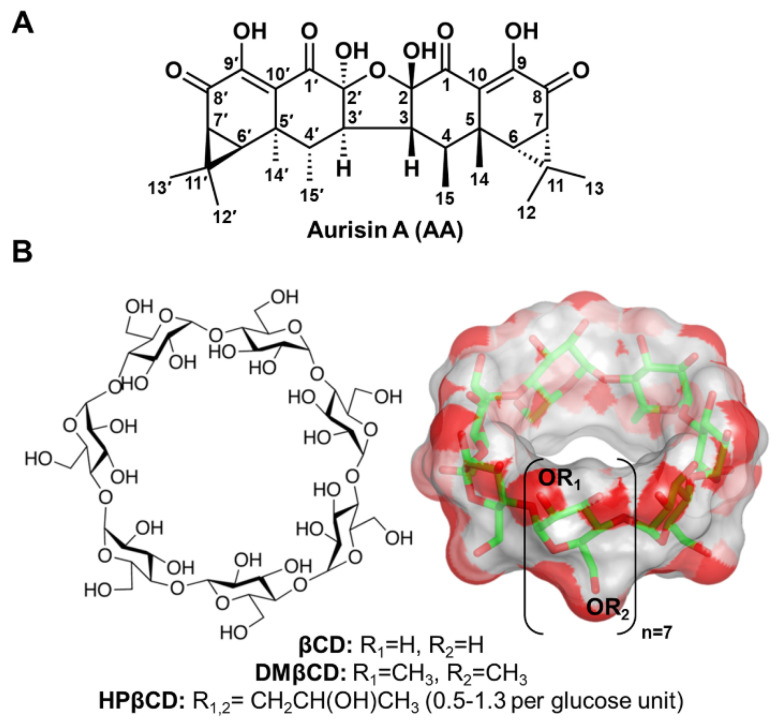
Chemical structures of (**A**) AA and (**B**) βCD and its derivatives (DMβCD and HPβCD), where the functional substitutions used in this study, are shown below.

Aurisin A (AA, Figure 1A) is an aristolane dimer sesquiterpene isolated from the luminescent mushroom *Neonothopanus nambi* Speg. (Marasmiaceae), which is normally found on logs or dead wood in broad-leaved forests in the northeast of Thailand [1]. This compound exhibits various biological and pharmacological activities, including antimycobacterial activity toward *Mycobacterium tuberculosis*, antimalarial property against *Plasmodium falciparum* [1], and anticancer potential toward lung cancer cells (NCI-H187 and A549) [1,2], breast cancer cells (BC1), epidermoid carcinoma cells (KB), cholangiocarcinoma cells (KKU-100, KKU-139, KKU-156, KKU-213, and KKU-214) [1], and cervical cancer cells (Hela, CaSki, and SiHa), with no cytotoxic effect on normal white blood cells [3]. AA exerts its anticancer effects by (i) inhibiting cancer cell growth and migration and (ii) inducing cell cycle arrest and apoptosis through activating caspase-3/9 as well as decreasing the expression of cyclin D1, cyclin-dependent kinase 2/4 (Cdk-2/4), B-cell lymphoma 2 (Bcl-2), epidermal growth factor receptor (EGFR), phosphorylated p38 (pp38), and vascular endothelial growth factor (VEGF) [2,3]. Although AA possesses promising biological and pharmacological activities, its low water solubility limits its use for further applications as herbal medicines or healthcare products.

Cyclodextrin (CD) is a cyclic oligosaccharide linked by α-1,4 glycosidic bonds. Natural CD consists of six, seven, and eight glucose units, namely alpha-cyclodextrin (αCD), beta-cyclodextrin (βCD), and gamma-cyclodextrin (γCD), respectively [4]. The structural arrangement of CD turns out to be a truncated cone shape structure possessing a hydrophilic outer surface with a hydrophobic inner cavity. By hosting lipophilic guest molecules into the central cavity of CD, their physico-chemical properties are tremendously improved [5,6,7], making CD the most frequently used excipient in pharmaceutical applications [8]. Among the three natural CDs, βCD (Figure 1B) has been widely used because of its suitable cavity size, commercial availability, desirable drug loading capacity, biocompatibility, and low price [9,10]. However, due to the limited solubility of βCD, derivatives of βCD, such as 2-hydroxypropyl-βCD (HPβCD) and 2,6-di-*O*-methyl-βCD (DMβCD), are developed to improve water solubility and reduce the limitations of the parent βCD [11,12]. Many lines of evidence have shown that the water solubility, chemical stability, and biological activity of poorly soluble compounds are significantly enhanced by complexation with βCD derivatives [13,14,15,16,17]. However, the information on the inclusion complexation between AA and βCD(s) has never been reported.

In the present study, we aimed to enhance the water solubility, stability, and anticancer activity of AA by inclusion complexation with βCD and its derivatives (DMβCD and HPβCD). The obtained inclusion complex was then confirmed experimentally and theoretically using physical and chemical characterization techniques as well as molecular modeling studies. In addition, the anticancer potential of the inclusion complex was evaluated. We hope that the improved physical and biological properties of AA/βCD(s) inclusion complex could pave the way for the further development of AA as herbal medicines or healthcare products.

## 2. Results and Discussion

### 2.1. Phase Solubility, Thermodynamic Parameters, and UV-Vis Spectra Analyses

**Figure 2:**
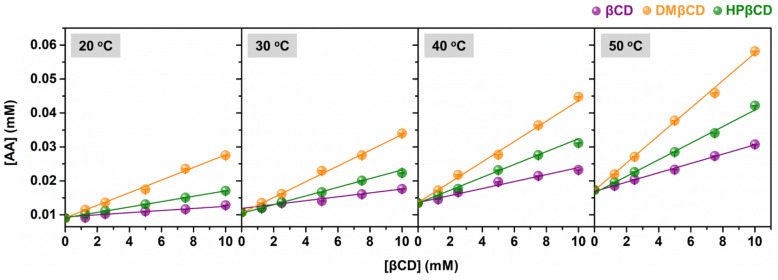
Phase solubility diagrams of AA in complex with βCD, DMβCD, and HPβCD at 20 °C, 30 °C, 40 °C, and 50 °C. Data are expressed as mean ± SEM of three independent experiments.

The phase solubility diagrams of AA in aqueous solutions of βCD, DMβCD, and HPβCD at 20, 30, 40, and 50 °C are shown in Figure 2. The obtained results revealed that the solubility of AA linearly increased with increasing concentrations of βCDs (ranked in the order of AA/DMβCD > AA/HPβCD > AA/βCD). This linear relationship is a characteristic of A_L_-type solubility, indicating a 1:1 host–guest complexation [18,19,20]. Next, the stability constant (*K_c_*) was calculated from the phase solubility diagrams to estimate the binding strength of all the studied inclusion complexes. As shown in Table 1, the highest *K_c_* value was found in AA/DMβCD (209–237 M^−1^), followed by AA/HPβCD (88–148 M^−1^) and AA/βCD (42–80 M^−1^), respectively. These findings are consistent with many lines of evidence demonstrating that βCD derivatives, especially DMβCD, could significantly improve the stability and solubility of several poorly soluble compounds [15,21,22,23]. Interestingly, the increased temperature remarkably enhanced the stability (the *K_c_* index) of all the investigated complexes.

To obtain the thermodynamic parameters (i.e., Δ*H*, Δ*S*, and Δ*G*) for the AA/βCD(s) inclusion complexation process, the Van’t Hoff plot based on Equation (3) was then employed (Figure S1). As depicted in Table 2, the Δ*H* values were positive for all systems, indicating that the inclusion complex formation was an endothermic process [14]. As expected, the inclusion complex formation between AA and βCD(s) was spontaneous, as evidenced by the negative sign of ∆*G*. The lowest Δ*G* value was detected in AA/DMβCD (−3.24 kcal/mol), followed by AA/HPβCD (−2.82 kcal/mol) and AA/βCD (−2.42 kcal/mol), respectively, which is consistent well with the aforementioned *K_c_* values (Table 1).

**Figure 3:**
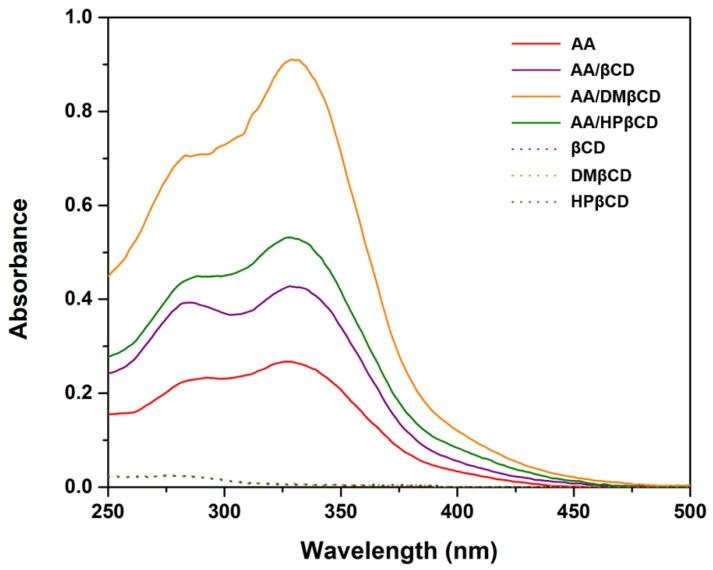
UV-Vis spectra of AA, inclusion complexes, and the free form of βCDs.

According to the UV-Vis spectra analysis (Figure 3), we found that the maximum absorption of AA (325 nm) bathochromically shifted to 328–332 nm in all the studied complexes, indicating a possible interaction between the AA and βCD(s). Similar bathochromic shifts of ligands after complexation were also found in luteolin and *trans*-ferulic acid in complex with the βCD derivatives [24,25]. Notably, the highest absorbance was detected in the AA/DMβCD complex (0.91), followed by AA/HPβCD (0.53), AA/βCD (0.43), and AA alone (0.27), which is consistent well with the phase solubility study mentioned above (Figure 2).

Taken together, only the AA/DMβCD complex possessing the highest stability and solubility was selected for further structural characterizations in comparison with the free form of AA and DMβCD.

### 2.2. Inclusion Complex Characterization

#### 2.2.1. FT-IR

**Figure 4:**
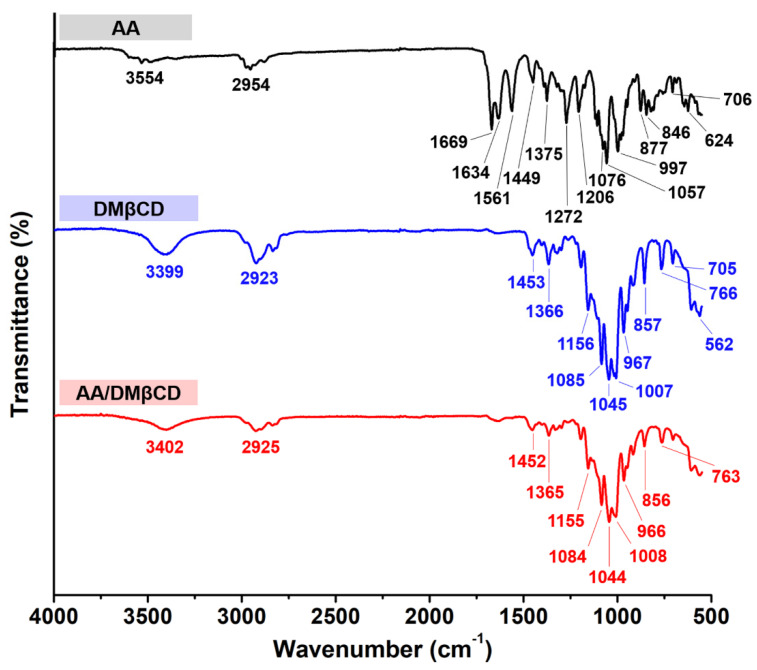
FT-IR spectra of the AA, DMβCD, and AA/DMβCD complex.

FT-IR spectroscopy was used to determine the inclusion complex formation between AA and DMβCD. The obtained FT-IR spectra of the AA, DMβCD, and AA/DMβCD complex are shown in Figure 4. The spectrogram of AA showed characteristic stretching vibration peaks at (i) 1669 and 1634 cm^−1^ for C=O, (ii) 1561 cm^−1^ for C=C, (iii) 2954 cm^−1^ for C−H, (iv) 1272 and 1206 cm^−1^ for C−O, and (v) 3554 cm^−1^ for O−H [1]. The FT-IR spectrum of DMβCD demonstrated a large band at 3399 cm^−1^ (O−H stretching), 2923 cm^−1^ (C−H stretching), and 1156, 1085, and 1045 cm^−1^ (C−O and C−H stretching) [26]. After the inclusion complexation, the FT-IR spectrum of AA/DMβCD was distinctly different from that of the pure AA and DMβCD. The characteristic stretching vibration peaks of AA at 1272, 1206, 1561, 1634, and 1669 cm^−1^ totally disappeared in the FT-IR spectrum of the inclusion complex, which is similar to other reported hydrophobic compounds in complex with the βCD analogs [14,25,27]. This might be due to a restriction of the AA’s C=O and C=C stretching vibrations, as well as a modification of the hydrophobic microenvironment inside the DMβCD cavity [28]. Moreover, the changes in the shape and position of the absorption bands of AA and DMβCD were observed in the AA/DMβCD complex. The vibration peaks of the C−H and O−H stretching of AA (2954 and 3554 cm^−1^) and DMβCD (2923 and 3399 cm^−1^) were shifted to 2925 and 3402 cm^−1^ in the solid complex. Similarly, the vibration peaks of the C−O and C−H stretching of DMβCD (1156, 1085, and 1045 cm^−1^) were redshifted to 1155, 1084, and 1044 cm^−1^ after the complex formation. Altogether, these FT-IR results indicated that AA was completely embedded in the hydrophobic cavity of DMβCD, which is supported by the differential scanning calorimetry (DSC), scanning electron microscope (SEM), and molecular dynamics (MD) simulation results, as discussed later.

#### 2.2.2. Thermal Analysis

**Figure 5:**
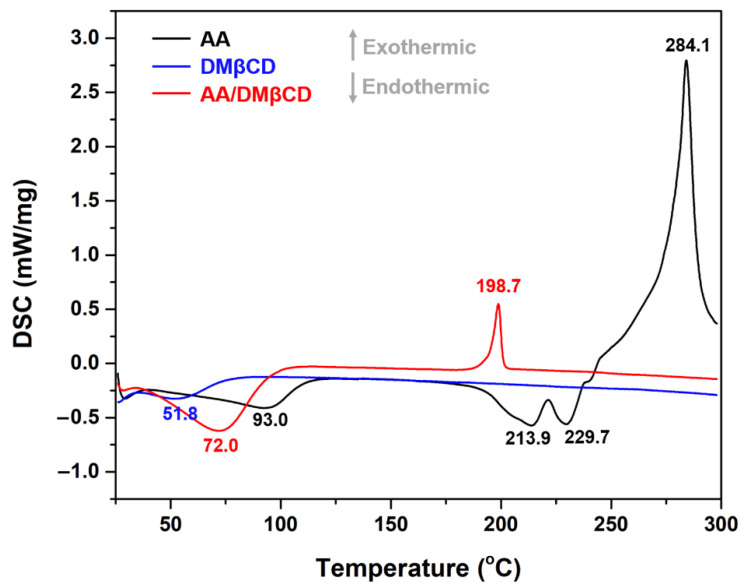
DSC thermograms of the AA, DMβCD, and AA/DMβCD complex.

The thermal properties of the AA, DMβCD, and AA/DMβCD complex were characterized in a solid state using DSC analysis. As shown in Figure 5, the characteristic endothermic/exothermic peaks of the free compounds were as follows: (i) AA at 93.0, 213.9, 229.7, and 284.1 °C and (ii) DMβCD at 51.8 °C. The endothermic peaks found at 213.9 and 229.7 °C corresponded to the melting point of AA, as previously reported [1], whereas the broad endothermic peak of DMβCD detected at 51.8 °C indicates the release of water molecules from the DMβCD’s hydrophobic inner cavity [29]. In the thermogram of the freeze-dried AA/DMβCD inclusion complex, the characteristic thermal peaks of AA and DMβCD totally disappeared, coinciding with the appearance of a new endothermic peak at 72.0 °C and an exothermic peak at 198.7 °C, similar to other reports [15,16,23,26,30]. These findings indicated that the freeze-drying method successfully yielded the new solid phase between AA and DMβCD.

#### 2.2.3. SEM

**Figure 6:**
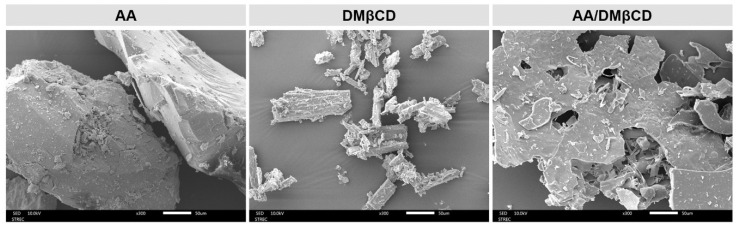
SEM photographs of the AA, DMβCD, and AA/DMβCD complex.

Many lines of evidence have shown that the inclusion complexation process significantly changes the surface textures of the resulting products [27,31,32,33]. To visualize the surface morphology of all the studied compounds, the SEM technique was employed. The SEM photographs of the AA, DMβCD, and AA/DMβCD complex are given in Figure 6. Both AA and DMβCD presented a rod-like structure [23,30]; however, the particle size and shape of the AA were bigger and more spherical than those of the DMβCD. Upon molecular complexation, the surface morphology of the obtained freeze-dried AA/DMβCD inclusion complex, appearing as a plate-like structure, was different from that of the pure forms. These findings confirmed the successful formation between AA and DMβCD. Taken together, all of the structural characterization results (Figure 4, Figure 5 and Figure 6) revealed that the inclusion complex between AA and DMβCD was successfully formed.

### 2.3. Molecular Modeling Studies

To further verify the aforementioned experimental results and to investigate the dynamic behavior of the AA/DMβCD inclusion complex at the atomic level, all-atom MD simulations in an aqueous solution and free energy calculations based on the molecular mechanics/Poisson–Boltzmann surface area (MM/PBSA) were performed.

#### 2.3.1. System Stability

**Figure 7:**
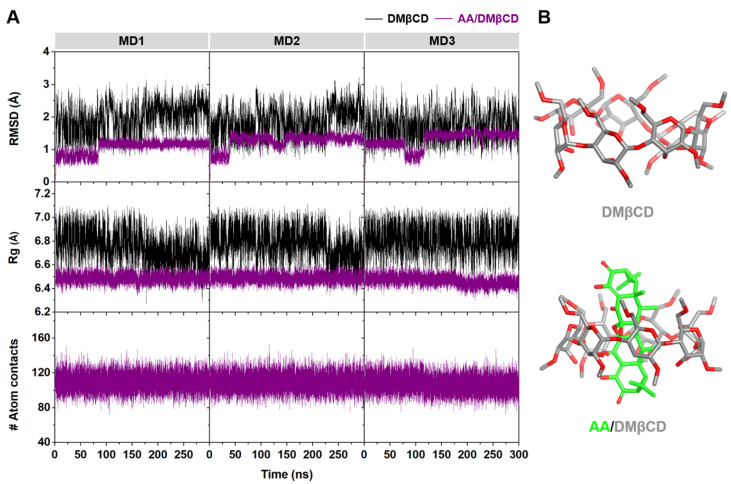
(**A**) Time evolution of RMSD, Rg, and # Atom contacts of DMβCD and AA/DMβCD for three independent simulations (MD1–3). (**B**) Final MD snapshot of DMβCD and AA/DMβCD.

The stability of DMβCD and its inclusion complex, AA/DMβCD, along the simulation time was determined using the calculated time evolution of root-mean-square displacement (RMSD), radius of gyration (Rg), and number of atomic contacts (# Atom contacts). As shown in Figure 7A, the RMSD values of DMβCD (~2–3 Å) were higher than those of the AA/DMβCD complex (~1 Å), suggesting that the AA/DMβCD is more stable than the uncomplexed DMβCD. Similarly, the Rg values of DMβCD (~6.6–7.0 Å) were larger than those of the AA/DMβCD complex (~6.4–6.6 Å), indicating higher compactness of the AA/DMβCD structure, as evidenced by the final MD snapshots (Figure 7B). Although the # Atom contacts (native + nonnative) was highly stable along the simulation times (~100–120) for the three independent simulations, the high RMSD fluctuations at the first 150 ns of the simulations were detected. Therefore, in this work, the MD trajectories from 200–300 ns were extracted for further structural and energetic analyses. From Figure 7B, we found that the AA molecule was completely embedded in the hydrophobic cavity of DMβCD, where the C=O groups of AA at C1 and C1′ (Figure 1A) were located at the center of DMβCD’s cavity, while those at C8 and C8′ were positioned near the secondary and primary rims of DMβCD. This complete formation of the AA/DMβCD complex is consistent well with the results of the inclusion complex characterization, as mentioned above.

#### 2.3.2. DMβCD Conformation upon AA Binding

**Figure 8:**
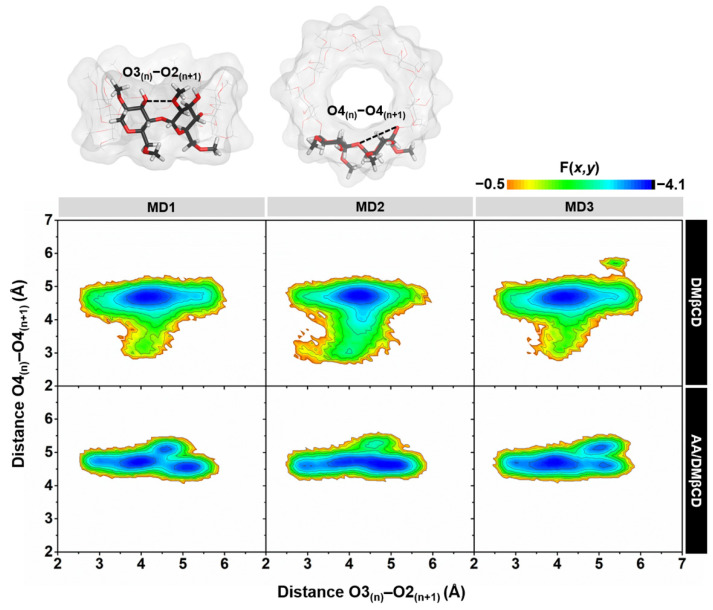
2D free energy (F(*x*,*y*)) landscape of the intramolecular hydrogen bond distances, O3_(n)_–O2_(n+1)_, against the adjacent glycosidic oxygen distances, O4_(n)_–O4_(n+1)_, of the DMβCD (**top**) and AA/DMβCD complex (**bottom**) for three independent simulations (MD1–3).

The conformational changes of DMβCD upon AA encapsulation were investigated by calculating (i) the distance of the oxygen atoms on the wider rim of DMβCD (O3_(n)_–O2_(n+1)_, dO_3-2_), corresponding to a possibility of an intramolecular hydrogen bond (H-bond) formation (dO_3-2_ ≤ 3.5 Å), and (ii) the distance of glycosidic oxygen atoms (O4_(n)_–O4_(n+1)_, dO_4-4_). Afterward, these two parameters were converted to the free energy value, F(*x*,*y*), using Equation (1):

F(*x*,*y*) = −*k*_B_*T* log[*P*(*x*,*y*)] (1)

where *k*_B_ is the Boltzmann constant, *T* is the temperature (303 K), and *P*(*x*,*y*) is the probability of dO_3-2_ (*x*) and dO_4-4_ (*y*). The obtained 2D free energy landscape is shown in Figure 8. When compared to the unbound form of DMβCD, the molecular encapsulation of AA in the DMβCD could enhance the formation of intramolecular H-bonds on the wider rim of DMβCD, as evidenced by the increased population of dO_3-2_ at ~3.0–3.5 Å. The H-bond-operated conformational changes of DMβCD upon the AA encapsulation are similar to other reported mansonones, pinostrobin, luteolin, pinocembrin, and neral in complex with various βCD derivatives [23,34,35,36,37]. In addition, the populations of dO_3-2_ at ~3.0–5.0 Å and dO_4-4_ at ~3.0–4.0 Å of the free form of DMβCD totally disappeared in the AA/DMβCD complex due to the adaptation of the DMβCD structure upon the insertion of AA to the hydrophobic cavity.

#### 2.3.3. Water Accessibility toward the Inclusion Complex

**Figure 9:**
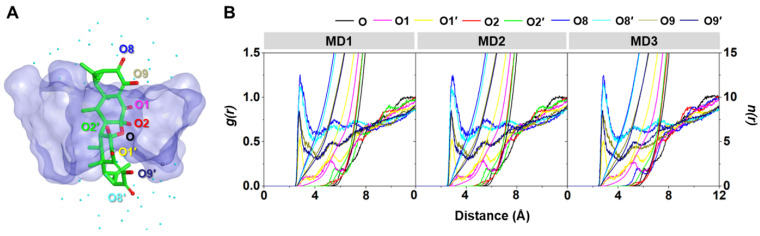
(**A**) Final MD snapshot of the AA/DMβCD complex showing the oxygen atoms (O, O1, O1′, O2, O2′, O8, O8′, O9, and O9′) of AA and the surrounding water molecules (cyan dot) within 5 Å of AA. (**B**) RDF of water oxygen atoms around the oxygen atoms of AA in complex with DMβCD for three independent simulations (MD1–3).

The water distribution around a spherical radius *r* of the oxygen atoms of AA (Figure 9A) was visualized using radial distribution function (RDF, *g*(*r*)) calculation, and the obtained results are given in Figure 9B. In addition, the integration number (*n*(*r*)) values at the first minima, corresponding to the number of water molecules approaching the targeted oxygens, are depicted in Table 3. From the RDF plots of all the systems, no dominant peak was detected within ~3 Å of the O, O1, O2, and O2′ of AA (Figure 9B), indicating that these oxygen atoms were deeply embedded in the hydrophobic inner cavity of DMβCD (Figure 9A). This phenomenon is in good agreement with the previously reported flavonoids, demonstrating that their oxygen atom on the chromone ring, embedded at the center of the βCD cavity, displayed no sharp RDF peak at the first solvation shell [36,38]. In contrast, the other oxygen atoms (O1′, O8, O8′, O9, and O9′) displayed the first sharp peak at ~2.5 Å, corresponding to the water distribution around these oxygens. The O8 and O8′ of AA exhibited higher water accessibility than the other oxygens, suggesting that these oxygen atoms were positioned nearby either the secondary or primary rim and were feasibly accessible to the water molecules (Figure 9A).

#### 2.3.4. Binding Affinity of the Inclusion Complex

To estimate the binding affinity of the AA/DMβCD inclusion complex, the MM/PBSA calculation was performed using 100 snapshots taken from the last 100 ns MD simulations. As expected, due to the poor solubility of AA, the inclusion complexation in the gas phase was driven mainly by van der Waals (vdW) interactions (∆*E*_vdW_ = −35.12 ± 1.06 kcal/mol) rather than electrostatic attraction (∆*E*_ele_ = −17.05 ± 0.19 kcal/mol). Similarly, the summation of ∆*G*_solv,non-polar_ + ∆*E*_vdW_ energies (−40.64 ± 1.12 kcal/mol) showed a negative value compared to that of ∆*G*_solv,polar_ + ∆*E*_ele_ energies (12.64 ± 0.28 kcal/mol), indicating that the vdW forces play an important role in the complex formation between AA and DMβCD in an aqueous environment. This vdW-driven host–guest complexation process is consistent well with other lipophilic ligands in complex with βCDs [39,40,41,42]. Notably, the predicted Δ*G*_bind,MM/PBSA_ value (−3.87 ± 0.68 kcal/mol) was almost identical to the experimental Δ*G* (Δ*G*_exp_, −3.24 kcal/mol) obtained from the Van’t Hoff plot (Table 4), suggesting the successful calculation of the AA/DMβCD complex.

### 2.4. DMβCD Enhances Cytotoxicity of AA against Lung Cancer Cells

**Figure 10:**
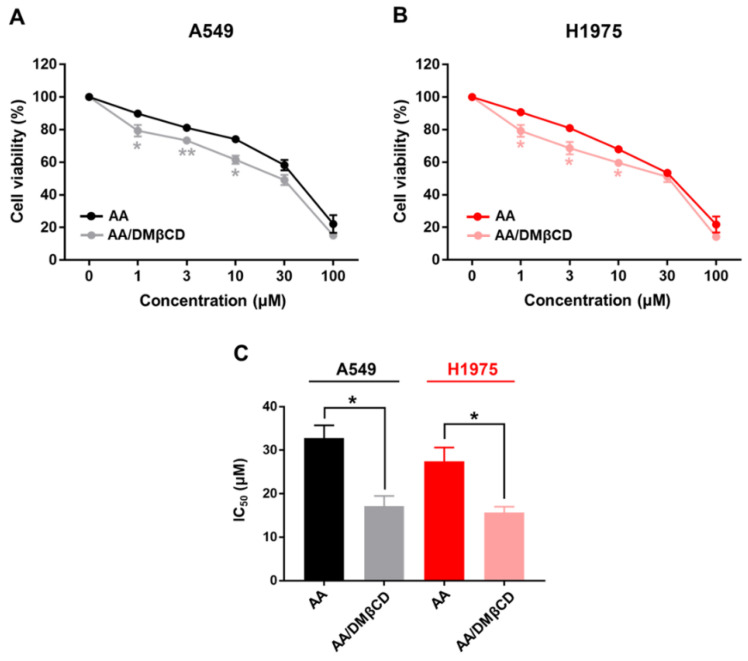
Cell viability of (**A**) A549 and (**B**) H1975 human lung cancer cells after being treated with various concentrations of AA and AA/DMβCD for 48 h. The viable cells in the vehicle control (0.2% DMSO) were calculated as 100%. (**C**) IC_50_ of the AA and AA/DMβCD complex against the A549 and H1975 cells. * *p* < 0.05, ** *p* < 0.01 vs. AA. Data are shown as mean ± SEM (*n* = 3).

The cytotoxic activity of AA and the AA/DMβCD inclusion complex against A549 and H1975 human lung cancer cells was evaluated using MTT assay. The obtained results are depicted in Figure 10. We found that both AA and AA/DMβCD decreased cell viability in a dose-dependent manner, in which the AA/DMβCD complex exhibited significantly lower cell viability than the uncomplexed AA at the concentration of 1, 3, and 10 μM for both A549 and H1975 cell lines (Figure 10A,B). The half-maximal inhibitory concentration (IC_50_) values against the A549 and H1975 cells of the AA/DMβCD inclusion complex (17.14 ± 2.34 and 15.67 ± 1.33 μM) were significantly lower than those of the AA alone (32.77 ± 2.94 and 27.38 ± 3.17 μM) (Figure 10C). These findings are in good agreement with previous reports demonstrating that βCDs can distinctly enhance the anticancer activity of several hydrophobic compounds, such as camptothecin, luotonin A, resveratrol, mansonone G, curcumin, and scutellarein [23,43,44,45,46]. Thus, it was assumed that the enhanced antitumor effect of the AA/DMβCD inclusion complex was due to the improved water solubility and complex stability (Figure 2 and Table 1). In addition, DMβCD could infiltrate into the drug permeation barrier, called the unstirred water layer (UWL) [47], better than the uncomplexed AA, enhancing the flux of AA through the UWL [48].

## 3. Materials and Methods

### 3.1. Materials

AA was extracted from the culture liquid of the luminescent mushroom *Neonothopanus nambi* PW1 (Marasmiaceae), as previously described [1,3]. βCD and HPβCD were purchased from TCI (Nihonbashi-honcho, CK, Tokyo), whereas DMβCD was purchased from Sigma-Aldrich (St. Louis, MO, USA).

### 3.2. Phase Solubility Study

The phase solubility study was performed according to the methods described by Higushi and Connors [49]. An excess amount of AA was added to aqueous solutions containing increasing amounts of βCD(s) (0–10 mM). The mixtures were incubated in a shaking incubator at 20, 30, 40, and 50 °C and 250 rpm for 40 h. After that, the insoluble AA was separated from the suspension by centrifugation at 10,000 rpm for 5 min and then filtered by a 0.45-micron syringe filter [13,23,44,50,51,52]. Two volumes of ethanol were added into each inclusion complex solution before measuring the absorbance at 331 nm [3]. The apparent stability constant (*K_c_*) was determined by Equation (2), where S_0_ is the y-intercept.

(2) $$K_{c}=\frac{\mathrm{Slope}}{S_{0}\left(1-\mathrm{slope}\right)}$$

The Van’t Hoff equation (Equation (3)) was used to calculate the change in the enthalpy (Δ*H*) and entropy (Δ*S*) of the inclusion complexation, whereas the Gibbs free energy (Δ*G*) was determined by Equation (4).

(3) $$\ln K_{c}=-\frac{\Delta H}{RT}+\frac{\Delta H}{R}$$

Δ*G* = Δ*H* − *T*Δ*S* (4)

### 3.3. Inclusion Complex Preparation

An excess amount of AA was added to a 10 mM DMβCD solution and incubated in a shaking incubator at 30 °C at 250 rpm. After that, the suspension was centrifuged (12,000 rpm for 15 min) and filtered through the 0.45-micron syringe filter, and lyophilized. The obtained freeze-dried powders were kept in a desiccator for further analysis.

### 3.4. Inclusion Complex Characterization

#### 3.4.1. Ultraviolet-Visible (UV-Vis) Spectroscopy

AA and its inclusion complexes were suspended in DI water at 30 °C for 48 h. After that, the suspension was filtered using the 0.45-micron syringe filter. The UV-Vis spectra of the solutions were recorded by Eppendorf BioSpectrometer™ (Eppendorf, Hamburgm, Germany).

#### 3.4.2. Fourier Transform Infrared (FT-IR) Spectroscopy

The FT-IR spectra of the AA, DMβCD, and AA/DMβCD complex were recorded by a Nicolet 6700 FT-IR spectrometer (ThemoFisher Scientific, Waltham, MA, USA) over a scanning range of 500–4000 cm^−1^ via the attenuated total reflectance (ATR) mode.

#### 3.4.3. Differential Scanning Calorimetry (DSC)

The thermal behavior of the AA, DMβCD, and AA/DMβCD complex was characterized using NETZSCH DSC 204F1 Phoenix (Selb, Germany). Each solid sample (~1–2 mg) was heated from 25 °C to 300 °C in aluminum pans at a rate of 10 °C/min.

#### 3.4.4. Scanning Electron Microscope (SEM)

The surface morphology of the AA, DMβCD, and AA/DMβCD complex was analyzed using a Scanning Electron Microscope (JEOL JSM-IT500HR, Tokyo, Japan). Samples were coated with a thin layer of gold in a vacuum before viewing under 300 times magnification. Observations were performed using an accelerating voltage of 10 kV.

### 3.5. Computational Details

#### 3.5.1. System Preparation and Molecular Docking

The 3D structure of DMβCD was taken from a previous study [23], whereas that of AA was downloaded from the PubChem database (PubChem CID: 71491081) and then optimized by the Gaussian09 program (Wallingford, CT, USA) [53] using the HF/6-31G* level of theory. The protonation state of AA was checked at a pH of 7.0 using MarvinSketch software (Budapest, Hungary). The inclusion complex model between the optimized AA and the DMβCD was generated using the CDOCKER module implemented in Accelrys Discovery Studio 2.5 (Accelrys Software Inc., San Diego, CA, USA). Among the resulting 100 docking poses, the AA/DMβCD inclusion complex with the lowest CDOCKER interaction energy was selected for further studies.

#### 3.5.2. Molecular Dynamics (MD) Simulations

The MD simulations with the isothermal-isobaric ensemble (*NPT*) of each system were performed with a time step of 2 fs using an AMBER16 software package [54]. According to the standard procedures [36,55,56], the electrostatic potential (ESP) charges of AA were calculated with the HF/6-31(d) level of theory using the antechamber module, whereas the restrained ESP (RESP) charges of AA were computed using the parmchk module in AMBER16. The SHAKE algorithm [57] was applied to constrain all chemical bonds involving hydrogen atoms, while the Particle Mesh Ewald [58] method was used to treat long-range electrostatic interactions. The cutoff value for non-bonded interactions was set to 12 Å. The general AMBER force field (GAFF) [59] and the Glycam-06 force field [60] were applied on AA and DMβCD, respectively. The TIP3P water molecules [61] were added to solvate the inclusion complex with a spacing distance of 15 Å. Subsequently, the water molecules were minimized using the steepest descent (1500 steps) and conjugated gradient (3000 steps), followed by the minimization of the whole system. Each studied system was heated up from 10 K to 298 K for 100 ps and then equilibrated for 1000 ps. After that, all-atom MD simulations were performed under a periodic boundary condition at 1 atm and 298 K until reaching 300 ns. The MD simulations were performed in three replicates (*n* = 3) for each model.

#### 3.5.3. Structural and Energetic Analyses

The CPPTRAJ module of AMBER16 was used to calculate the structural information, including the RMSD, Rg, # Atom contacts, free energy landscape, and RDF. For the energetic analysis, the binding affinity between the host and guest was calculated by the MM/PBSA method [62] using 100 snapshots extracted from the last 100 ns MD simulations.

### 3.6. Cell Lines and Culture

A549 and H1975 human lung cancer cells were purchased from the American Type Culture Collection (ATCC, Manassas, VA, USA). Both cells were cultured in a Dulbecco’s Modified Eagle’s Medium (Gibco, NY, USA) supplemented with a 10% heat-inactivated fetal bovine serum (Gibco, NY, USA), 100 U/mL penicillin, and 100 µg/mL streptomycin (Gibco, NY, USA) and were maintained at 37 °C in a humidified 5% CO_2_ atmosphere.

### 3.7. Cell Viability Assay

The A549 and H1975 cells were seeded into 96-well plates at a density of 1000 cells/well. After overnight incubation, the cells were treated with logarithmic concentrations (1, 3, 10, 30, and 100 µM) of AA and AA/DMβCD for 48 h. Note that the amount of MG in free form and in complex form was equivalent. An MTT reagent was then added to the wells and incubated for 3 h. Subsequently, the culture medium was withdrawn, and 100 µL of a DMSO solution was added to dissolve the formazan crystals. Finally, the absorbance was measured at 540 nm.

### 3.8. Statistical Analysis

Data are shown as mean ± standard error of the mean (SEM) of three independent experiments. Differences between AA and AA/DMβCD were determined using the *t* test. A *p* value of <0.05 was considered statistically significant.

## 4. Conclusions

This study aimed to improve the water solubility and biological activity of AA by complexation with βCD and its derivatives (DMβCD and HPβCD). The phase solubility diagrams indicated 1:1 AA/βCD(s) binding stoichiometry, and the highest *K_c_* was detected in the AA/DMβCD complex. Notably, βCDs, especially DMβCD, increased the thermal stability of the complexes. The thermodynamic study indicated that the inclusion complexation between AA and βCD(s) was a spontaneous endothermic reaction. The complex formation of the AA/DMβCD was confirmed by UV-Vis, FT-IR, DSC, and SEM techniques. MD simulations and MM/PBSA-based free energy calculations affirmed the vdW-driven formation of the AA/DMβCD complex in an aqueous environment. The anticancer effect of AA on A549 and H1975 lung cancer cells was significantly improved by complexation with DMβCD. Taken together, the satisfactory water solubility, high thermal stability, and enhanced antitumor potential of the AA/DMβCD complex would be potentially useful for its application as herbal medicines or healthcare products.

## Figures and Tables

**Table 1:** Stability constant (*K_c_*) of the AA/βCDs inclusion complexes at different temperatures.

Temperature (°C)	*K_c_* (M^−1^)		
	AA/βCD	AA/DMβCD	AA/HPβCD
20	42	209	88
30	60	219	112
40	73	232	135
50	80	237	148

**Table 2:** Thermodynamic values for the inclusion complexation between AA and βCD(s).

Thermodynamic Parameters (kcal/mol) ^a^	AA/βCD	AA/DMβCD	AA/HPβCD
Δ*H*	4.05	0.84	3.32
TΔ*S*	6.47	4.08	6.14
Δ*G*	−2.42	−3.24	−2.82

^a^ Data were derived from Van’t Hoff plots using R = 1.985 × 10^−3^ kcal·mol^−1^·K^−1^ and T = 303 K.

**Table 3:** *n*(*r*) up to the first minimum for the oxygen atoms of AA inside the DMβCD hydrophobic cavity.

	O	O1	O1′	O2	O2′	O8	O8′	O9	O9′
*n*(*r*) ^a^	-	-	0.96 ± 0.02	-	-	5.77 ± 0.43	4.74 ± 0.18	4.15 ± 0.16	3.07 ± 0.11

^a^ Data derived from Figure 9 are shown as mean ± SEM (*n* = 3).

**Table 4:** ∆*G*_bind, MM/PBSA_ and its energy components (kcal/mol) of the AA/DMβCD complex.

Energy Component (kcal/mol) ^a^	AA/DMβCD
Δ*E*_vdW_	−35.12 ± 1.06
Δ*E*_ele_	−17.05 ± 0.19
Δ*E*_MM_	−52.18 ± 0.92
Δ*G*_solv,polar_	29.69 ± 0.16
Δ*G*_solv,non-polar_	−5.51 ± 0.06
Δ*G*_solv_	24.18 ± 0.19
∆*G*_solv,polar_ + ∆*E*_ele_	12.64 ± 0.28
∆*G*_solv,non-polar_ + ∆*E*_vdW_	−40.64 ± 1.12
TΔ*S*	−24.12 ± 0.16
Δ*G*_bind,MM/PBSA_	−3.87 ± 0.68
Δ*G*_exp_^b^	−3.24

^a^ Data are shown as mean ± SEM (*n* = 3). ∆*E*_MM_, molecular mechanics energy; ∆*G*_solv_, solvation free energy comprising polar (∆*G*_solv,polar_) and non-polar (∆*G*_solv,non-polar_) terms; ∆*S*, entropy. ^b^ Data from Table 2.

## Data Availability

Data is contained within the article or Supplementary Material.

## Supplementary Materials

The supporting information can be downloaded at: https://www.mdpi.com/article/10.3390/ijms23179776/s1.

## References

[1] Kanokmedhakul S., Lekphrom R., Kanokmedhakul K., Hahnvajanawong C., Bua-art S., Saksirirat W., Prabpai S., Kongsaeree P. (2012). Cytotoxic sesquiterpenes from luminescent mushroom Neonothopanus nambi. Tetrahedron.

[2] Boueroy P., Boonmars T., Kanokmedhakul S., Chareonsudjai S., Lekphrom R., Srichangwang S. (2020). Promising Anticancer Effect of Aurisin A Against the Human Lung Cancer A549 Cell Line. Asian Pac. J. Cancer Prev..

[3] Boueroy P., Kanokmedhakul S., Pientong C., Ekalaksananan T., Saksirirat W., Ratanasuwan P., Lekphrom R., Srichangwang S. (2021). Anticancer effects of aurisin A extracts from Neonothopanus nambi on human papillomavirus-infected cervical cancer cells. Agric. Nat. Resour..

[4] Szejtli J. (1998). Introduction and general overview of cyclodextrin chemistry. Chem. Rev..

[5] Soe H.M.H., Chamni S., Mahalapbutr P., Kongtaworn N., Rungrotmongkol T., Jansook P. (2020). The investigation of binary and ternary sulfobutylether-β-cyclodextrin inclusion complexes with asiaticoside in solution and in solid state. Carbohydr. Res..

[6] Jiang L., Yang J., Wang Q., Ren L., Zhou J. (2019). Physicochemical properties of catechin/β-cyclodextrin inclusion complex obtained via co-precipitation. CyTA-J. Food.

[7] Calabrò M.L., Tommasini S., Donato P., Raneri D., Stancanelli R., Ficarra P., Ficarra R., Costa C., Catania S., Rustichelli C. (2004). Effects of α- and β-cyclodextrin complexation on the physico-chemical properties and antioxidant activity of some 3-hydroxyflavones. J. Pharm. Biomed. Anal..

[8] Conceição J., Adeoye O., Cabral-Marques H.M., Lobo J.M.S. (2018). Cyclodextrins as excipients in tablet formulations. Drug Discov. Today.

[9] Del Valle E.M.M. (2004). Cyclodextrins and their uses: A review. Process Biochem..

[10] Tommasini S., Raneri D., Ficarra R., Calabrò M.L., Stancanelli R., Ficarra P. (2004). Improvement in solubility and dissolution rate of flavonoids by complexation with β-cyclodextrin. J. Pharm. Biomed..

[11] Gould S., Scott R.C. (2005). 2-Hydroxypropyl-β-cyclodextrin (HP-β-CD): A toxicology review. Food Chem. Toxicol..

[12] Szejtli J. (1983). Dimethyl-β-cyclodextrin as parenteral drug carrier. J. Incl. Phenom..

[13] Kicuntod J., Sangpheak K., Mueller M., Wolschann P., Viernstein H., Yanaka S., Kato K., Chavasiri W., Pongsawasdi P., Kungwan N. (2018). Theoretical and Experimental Studies on Inclusion Complexes of Pinostrobin and β-Cyclodextrins. Sci. Pharm..

[14] Tang P., Li S., Wang L., Yang H., Yan J., Li H. (2015). Inclusion complexes of chlorzoxazone with β- and hydroxypropyl-β-cyclodextrin: Characterization, dissolution, and cytotoxicity. Carbohydr. Polym..

[15] Wang L., Li S., Tang P., Yan J., Xu K., Li H. (2015). Characterization and evaluation of synthetic riluzole with β-cyclodextrin and 2,6-di-O-methyl-β-cyclodextrin inclusion complexes. Carbohydr. Polym..

[16] Yang L.-J., Ma S.-X., Zhou S.-Y., Chen W., Yuan M.-W., Yin Y.-Q., Yang X.-D. (2013). Preparation and characterization of inclusion complexes of naringenin with β-cyclodextrin or its derivative. Carbohydr. Polym..

[17] Oo A., Kerdpol K., Mahalapbutr P., Rungrotmongkol T. (2022). Molecular encapsulation of emodin with various β-cyclodextrin derivatives: A computational study. J. Mol. Liq..

[18] Liu M., Dong L., Chen A., Zheng Y., Sun D., Wang X., Wang B. (2013). Inclusion complexes of quercetin with three β-cyclodextrins derivatives at physiological pH: Spectroscopic study and antioxidant activity. Spectrochim. Acta Part A Mol. Biomol. Spectrosc..

[19] Santos P.S., Souza L.K.M., Araújo T.S.L., Medeiros J.V.R., Nunes S.C.C., Carvalho R.A., Pais A.C.C., Veiga F.J.B., Nunes L.C.C., Figueiras A. (2017). Methyl-β-cyclodextrin Inclusion Complex with β-Caryophyllene: Preparation, Characterization, and Improvement of Pharmacological Activities. ACS Omega.

[20] Gao S., Bie C., Ji Q., Ling H., Li C., Fu Y., Zhao L., Ye F. (2019). Preparation and characterization of cyanazine–hydroxypropyl-beta-cyclodextrin inclusion complex. RSC Adv..

[21] Kikuchi M., Uekama K. (1988). Effect of dimethyl beta-cyclodextrin on oral or rectal absorption of 1-hexylcarbamoyl-5-fluorouracil (HCFU). Yakugaku Zasshi J. Pharm. Soc. Jpn..

[22] Tang P., Wang L., Ma X., Xu K., Xiong X., Liao X., Li H. (2017). Characterization and In Vitro Evaluation of the Complexes of Posaconazole with β- and 2,6-di-O-methyl-β-cyclodextrin. AAPS PharmSciTech.

[23] Mahalapbutr P., Wonganan P., Charoenwongpaiboon T., Prousoontorn M., Chavasiri W., Rungrotmongkol T. (2019). Enhanced Solubility and Anticancer Potential of Mansonone G By β-Cyclodextrin-Based Host-Guest Complexation: A Computational and Experimental Study. Biomolecules.

[24] Liu B., Li W., Zhao J., Liu Y., Zhu X., Liang G. (2013). Physicochemical characterisation of the supramolecular structure of luteolin/cyclodextrin inclusion complex. Food Chem..

[25] Wang J., Cao Y., Sun B., Wang C. (2011). Characterisation of inclusion complex of trans-ferulic acid and hydroxypropyl-β-cyclodextrin. Food Chem..

[26] Wang D.-W., Ouyang C.-B., Liu Q., Yuan H.-L., Liu X.-H. (2013). Inclusion of quinestrol and 2,6-di-O-methyl-β-cyclodextrin: Preparation, characterization, and inclusion mode. Carbohydr. Polym..

[27] Rajendiran N., Siva S. (2014). Inclusion complex of sulfadimethoxine with cyclodextrins: Preparation and characterization. Carbohydr. Polym..

[28] Vukic M.D., Vukovic N.L., Popovic S.L., Todorovic D.V., Djurdjevic P.M., Matic S.D., Mitrovic M.M., Popovic A.M., Kacaniova M.M., Baskic D.D. (2020). Effect of β-cyclodextrin encapsulation on cytotoxic activity of acetylshikonin against HCT-116 and MDA-MB-231 cancer cell lines. Saudi Pharm. J. SPJ Off. Publ. Saudi Pharm. Soc..

[29] Gu F., Ning J., Fan H., Wu C., Wang Y. (2018). Preparation and characterization of simvastatin/DMβCD complex and its pharmacokinetics in rats. Acta Pharm..

[30] Choi S.G., Lee S.-E., Kang B.-S., Ng C.L., Davaa E., Park J.-S. (2014). Thermosensitive and Mucoadhesive Sol-Gel Composites of Paclitaxel/Dimethyl-β-Cyclodextrin for Buccal Delivery. PLoS ONE.

[31] Rakmai J., Cheirsilp B., Mejuto J.C., Simal-Gándara J., Torrado-Agrasar A. (2018). Antioxidant and antimicrobial properties of encapsulated guava leaf oil in hydroxypropyl-beta-cyclodextrin. Ind. Crops Prod..

[32] Yallapu M.M., Jaggi M., Chauhan S.C. (2010). β-Cyclodextrin-curcumin self-assembly enhances curcumin delivery in prostate cancer cells. Colloids Surf. B Biointerfaces.

[33] Zhou Q., Wei X., Dou W., Chou G., Wang Z. (2013). Preparation and characterization of inclusion complexes formed between baicalein and cyclodextrins. Carbohydr. Polym..

[34] Mahalapbutr P., Nutho B., Wolschann P., Chavasiri W., Kungwan N., Rungrotmongkol T. (2018). Molecular insights into inclusion complexes of mansonone E and H enantiomers with various β-cyclodextrins. J. Mol. Graph. Model..

[35] Kicuntod J., Khuntawee W., Wolschann P., Pongsawasdi P., Chavasiri W., Kungwan N., Rungrotmongkol T. (2016). Inclusion complexation of pinostrobin with various cyclodextrin derivatives. J. Mol. Graph. Model..

[36] Mahalapbutr P., Thitinanthavet K., Kedkham T., Nguyen H., Theu L.t.h., Dokmaisrijan S., Huynh L., Kungwan N., Rungrotmongkol T. (2019). A theoretical study on the molecular encapsulation of luteolin and pinocembrin with various derivatized beta-cyclodextrins. J. Mol. Struct..

[37] Wongpituk P., Nutho B., Panman W., Kungwan N., Wolschann P., Rungrotmongkol T., Nunthaboot N. (2017). Structural dynamics and binding free energy of neral-cyclodextrins inclusion complexes: Molecular dynamics simulation. Mol. Simul..

[38] Choi Y., Lee J., Kum W.C., Hwang S., Jeong K., Jung S. (2005). Binding geometry of inclusion complex as a determinant factor for aqueous solubility of the flavonoid/β-cyclodextrin complexes based on molecular dynamics simulations. Bull. Korean Chem. Soc..

[39] Chen W., Chang C.E., Gilson M.K. (2004). Calculation of cyclodextrin binding affinities: Energy, entropy, and implications for drug design. Biophys. J..

[40] Liu L., Guo Q.-X. (2002). The Driving Forces in the Inclusion Complexation of Cyclodextrins. J. Incl. Phenom. Macrocycl. Chem..

[41] Fermeglia M., Ferrone M., Lodi A., Pricl S. (2003). Host–guest inclusion complexes between anticancer drugs and β-cyclodextrin: Computational studies. Carbohydr. Polym..

[42] Alvira E. (2018). Theoretical Study of the β-Cyclodextrin Inclusion Complex Formation of Eugenol in Water. Molecules.

[43] González-Ruiz V., Cores Á., Martín-Cámara O., Orellana K., Cervera-Carrascón V., Michalska P., Olives A.I., León R., Martín M.A., Menéndez J.C. (2021). Enhanced Stability and Bioactivity of Natural Anticancer Topoisomerase I Inhibitors through Cyclodextrin Complexation. Pharmaceutics.

[44] Hao X., Sun X., Zhu H., Xie L., Wang X., Jiang N., Fu P., Sang M. (2021). Hydroxypropyl-β-Cyclodextrin-Complexed Resveratrol Enhanced Antitumor Activity in a Cervical Cancer Model: In Vivo Analysis. Front. Pharmacol..

[45] Zhang L., Man S., Qiu H., Liu Z., Zhang M., Ma L., Gao W. (2016). Curcumin-cyclodextrin complexes enhanced the anti-cancer effects of curcumin. Environ. Toxicol. Pharmacol..

[46] Wang F., Yang B., Zhao Y., Liao X., Gao C., Jiang R., Han B., Yang J., Liu M., Zhou R. (2014). Host-guest inclusion system of scutellarein with 2-hydroxypropyl-beta-cyclodextrin: Preparation, characterization, and anticancer activity. J. Biomater. Sci. Polym. Ed..

[47] Loftsson T. (2012). Drug permeation through biomembranes: Cyclodextrins and the unstirred water layer. Pharmazie.

[48] Brewster M.E., Noppe M., Peeters J., Loftsson T. (2007). Effect of the unstirred water layer on permeability enhancement by hydrophilic cyclodextrins. Int. J. Pharm..

[49] Higuchi T. (1965). A phase solubility technique. Adv. Anal. Chem. Instrum..

[50] Chierentin L., Garnero C., Chattah A.K., Delvadia P., Karnes T., Longhi M.R., Salgado H.R. (2015). Influence of β-cyclodextrin on the Properties of Norfloxacin Form A. AAPS PharmSciTech.

[51] Lopalco A., Manni A., Keeley A., Haider S., Li W., Lopedota A., Altomare C.D., Denora N., Tuleu C. (2022). In Vivo Investigation of (2-Hydroxypropyl)-β-cyclodextrin-Based Formulation of Spironolactone in Aqueous Solution for Paediatric Use. Pharmaceutics.

[52] Piras A.M., Fabiano A., Chiellini F., Zambito Y. (2018). Methyl-β-cyclodextrin quaternary ammonium chitosan conjugate: Nanoparticles vs. macromolecular soluble complex. Int. J. Nanomed..

[53] Frisch M.J., Trucks G.W., Schlegel H.B., Scuseria G.E., Robb M.A., Cheeseman J.R., Scalmani G., Barone V., Petersson G.A., Nakatsuji H. (2009). Gaussian 09.

[54] Case D.A., Betz R.M., Cerutti D.S., Cheatham T., Darden T.A., Duke R.E., Giese T.J., Gohlke H., Goetz A.W., Homeyer N. (2016). Amber 2016.

[55] Kerdpol K., Kicuntod J., Wolschann P., Mori S., Rungnim C., Kunaseth M., Okumura H., Kungwan N., Rungrotmongkol T. (2019). Cavity Closure of 2-Hydroxypropyl-β-Cyclodextrin: Replica Exchange Molecular Dynamics Simulations. Polymers.

[56] Mahalapbutr P., Sangkhawasi M., Kammarabutr J., Chamni S., Rungrotmongkol T. (2020). Rosmarinic Acid as a Potent Influenza Neuraminidase Inhibitor: In Vitro and In Silico Study. Curr. Top. Med. Chem..

[57] Ryckaert J.-P., Ciccotti G., Berendsen H.J.C. (1977). Numerical integration of the cartesian equations of motion of a system with constraints: Molecular dynamics of n-alkanes. J. Comput. Phys..

[58] Luty B.A., van Gunsteren W.F. (1996). Calculating Electrostatic Interactions Using the Particle−Particle Particle−Mesh Method with Nonperiodic Long-Range Interactions. Am. J. Phys. Chem..

[59] Wang J., Wolf R.M., Caldwell J.W., Kollman P.A., Case D.A. (2004). Development and testing of a general amber force field. J. Comput. Chem..

[60] Kirschner K.N., Yongye A.B., Tschampel S.M., González-Outeiriño J., Daniels C.R., Foley B.L., Woods R.J. (2008). GLYCAM06: A generalizable biomolecular force field. Carbohydrates. J. Comput. Chem..

[61] Jorgensen W.L., Chandrasekhar J., Madura J.D., Impey R.W., Klein M.L. (1983). 83 Comparison of simple potential functions for simulating liquid water. J. Chem. Phys..

[62] Genheden S., Ryde U. (2015). The MM/PBSA and MM/GBSA methods to estimate ligand-binding affinities. Expert Opin. Drug Discov..

