# Supplementary material for: Aurisin A Complexed with 2,6-Di-O-methyl-β-cyclodextrin Enhances Aqueous Solubility, Thermal Stability, and Antiproliferative Activity against Lung Cancer Cells
Source: Int J Mol Sci. 2022 Aug 29;23(17):9776. doi: 10.3390/ijms23179776 (PMC9456185; doi:10.3390/ijms23179776)
Supplement: Supplementary file 1 [file ijms-23-09776-s001.zip › ijms-1851553-supplementary.pdf]

Supplementary data

Aurisin A Complexed with 2,6-di-*O*-methyl-β-cyclodextrin Enhances Aqueous Solubility, Thermal Stability, and Antiproliferative Activity against Lung Cancer Cells

Thanapon Charoenwongpaiboon [1], Amy Oo [2], Sutita Nasoontorn [3], Thanyada Rungrotmongkol [2,4], Somdej Kanokmedhakul [5] and Panupong Mahalapbutr [3,*]

1 Department of Chemistry, Faculty of Science, Silpakorn University, Nakhon Pathom 73000, Thailand
2 Center of Excellence in Structural and Computational Biology, Department of Biochemistry, Chulalongkorn University, Bangkok 10330, Thailand
3 Department of Biochemistry, Center for Translational Medicine, Faculty of Medicine, Khon Kaen University, Khon Kaen 40002, Thailand
4 Ph.D. Program in Bioinformatics and Computational Biology, Graduate School, Chulalongkorn University, Bangkok 10330, Thailand
5 Natural Products Research Unit, Department of Chemistry and Center for Innovation in Chemistry, Faculty of Science, Khon Kaen University, Khon Kaen 40002, Thailand
* Correspondence: panupma@kku.ac.th

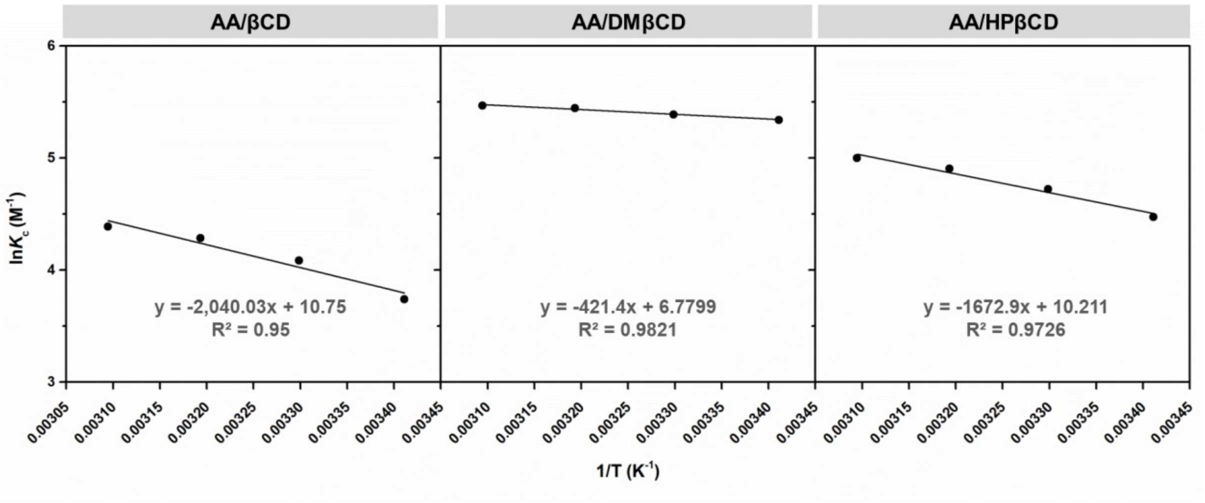

Figure S1. Van't Hoff plots of AA/βCDs inclusion complexes.
